# Balancing supply and demand in the presence of renewable generation via demand response for electric water heaters

**DOI:** 10.1007/s10479-020-03580-1

**Published:** 2020-03-31

**Authors:** Adham I. Tammam, Miguel F. Anjos, Michel Gendreau

**Affiliations:** 1 GERAD and Department of Mathematics and Industrial Engineering, Polytechnique Montreal, C.P. 6079, Succ. Centre-Ville, Montreal, QC H3C 3A7 Canada; 2 School of Mathematics, University of Edinburgh, James Clerk Maxwell Building, The King’s Buildings, Peter Guthrie Tait Road, Edinburgh, EH9 3FD United Kingdom; 3 CIRRELT and Department of Mathematics and Industrial Engineering, Polytechnique Montreal, C.P. 6079, Succ. Centre-Ville, Montreal, QC H3C 3A7 Canada

**Keywords:** Demand response, Electric water heaters, Stochastic optimization, Renewable power generation

## Abstract

With the increasing presence of renewable energy sources in the electrical power grid, demand response via thermostatic appliances such as electric water heaters is a promising way to compensate for the significant variability in renewable power generation. We propose a multistage stochastic optimization model that computes the optimal day-ahead target profile of the mean thermal energy contained in a large population of heaters, given various possible wind power production and uncontrollable load scenarios. This optimal profile is calculated to make the variable net demand as even as possible.

## Introduction

Compared to thermal resources, renewable energy sources are expensive in terms of equipment, installation, and maintenance. Their increasing use in electric grids is mainly due to the desire to reduce greenhouse gas emissions from burning fossil fuels. The variability of renewables creates challenges for system operators who must ensure a balance between supply (constrained by ramping limits) and demand. A promising tool to provide means to cope with these challenges is the provision of flexibility by the demand from the loads in the system. In particular, the load curve of thermostatically controlled appliances (TCAs) such as electric water heaters (EWHs), space heaters, and the batteries of connected electric vehicles, can in principle be reshaped while respecting the end-user comfort constraints.

The work in this paper was carried out as part of the smartDESC (smart Distributed Energy Storage Controller) project (Sirois et al. 2017). The objective of this project was to develop and validate a scalable methodology to harness the energy potential of very large numbers of small TCAs distributed throughout an electrical grid. The resulting methodology provides a tool that can be used for traditional peak shaving as well as to reduce the impacts of the fluctuations of intermittent renewable energy sources, particularly solar and wind.

The smartDESC project focused on controlling the load of EWHs, which can store energy for considerable periods because of their high thermal inertia. Their demand peak coincides with the peak of the total demand, so a significant reduction of the load curve peak could potentially be achieved (Sepulveda et al. 2010). According to Natural Resources Canada, the power consumption of EWHs in Canada can reach 21.7% of the total demand (Energy Use Data Handbook 2004). With renewable generation capacity growing quickly in Canada, notably with wind capacity doubling by 2040 (Canada’s Energy Future 2017), EWHs as a balancing resource is only going to grow in importance.

More generally, the demand-side management of TCAs is a promising way to counterbalance the variability of renewables, and it has been the focus of many studies. Dynamic programming models have been developed to minimize the peak load given a deterministic demand (Bischke and Sella 1985; Cohen and Wang 1988; Zhang and Li 2013). A control algorithm (Kondoh et al. 2011) has been developed to allow TCAs to follow regulation signals in order to stabilize a network supplied with renewable resources. A fuzzy logic control strategy (Nehrir and LaMeres 2000; LaMeres et al. 1999) can be used for TCA load shifting from peak to off-peak periods.

Lee and Wilkins (1983) have proposed a deterministic linear optimization model that decides the number of EWHs to which a control scheme selected from a predefined set should be applied in order to reduce the peak load. In Laurent et al. (1995), a column generation approach is applied to a load management problem where the objective is to minimize the maximum peak of a known load profile by choosing from a large set of admissible interruption scenarios established in advance. A metaheuristic algorithm based on particle swarm optimization to manage the power consumption of EWHs has been proposed (Rosario et al. 2011; Sepulveda et al. 2010). Directly controlling the power consumption of EWHs could reduce power losses in an electric grid as in Salehfar and Wehbe (2001).

The aforementioned studies consider a deterministic setting, but in reality the load demand and renewable supply are uncertain. Malik and Havel (2014) proposed centralized direct load control of EWHs to reduce the peak imports and exports in the Czech electricity market. Their approach takes into account the stochastic nature of load demand and renewable production. The dispatch of the EWHs is decided through a two-stage stochastic optimization program, where the first stage computes the overall EWH load, and the second stage adjusts the dispatch according to the actual power supply and demand.

The geographical distribution of the EHWs, and of distributed energy resources in general, is an important practical concern. Multiple challenges arise in the control of a large number of small but diverse storage devices spread over a wide area. A control schedule applied indiscriminately can reduce their natural load diversity, inducing the payback phenomenon that may create new peak loads (Laurent et al. 1995). Moreover, carefully controlling a large population of storage devices requires sophisticated mathematical models and significant computational power.

For these reasons, the methodology proposed in smartDESC combines a stochastic optimization model and a mean field model. More specifically, we consider a two-phase approach to the control problem. In the first phase, we optimally schedule the day-ahead load of a homogeneous aggregated model of the EWH population. The objective is to even out the net load as much as possible. There are two stochastic parameters: (1) the uncontrollable load (the total load after excluding the controllable EWH demand) and (2) the renewable supply. We use a stochastic optimization model to compute an optimal power profile (OPP) for this aggregated model. This profile is then translated into a temperature profile that specifies a series of hourly setpoints that the hot water as an aggregate is required to reach, in order to achieve the OPP. In the second phase, a local control module sends instructions to individual EWHs to ensure that the mean thermal energy of the EWH population follows the OPP as closely as possible. This local controller (Kizilkale and Malhame 2014) is based on mean field theory; we refer to it as a mean field controller (MFC). This paper is concerned with the first phase. We refer the reader to (Kizilkale and Malhame 2014; Kizilkale and Malhamé 2013) for more information on the second phase.

The contribution of this paper is firmly set in the context described above. We propose a multistage stochastic optimization model called the *Scheduler* that, given the current state of the EHWs, as well as information on the total demand and the wind production for the next *T* time periods, computes the OPP that minimizes the mean variation of the net demand over those *T* time periods. The optimal solution is then sent to the MFC to be applied via instructions to each individual EHW. A key point here is that the signals sent to the EWHs are in terms of temperature, which is a proxy for energy, but the desired effect is quantified in terms of power shifted from certain periods of time to others to achieve the reduction of variation of the net demand.

It is important to note that in the context of smartDESC, the stochastic optimization problem is solved on a rolling horizon basis. In other words, at each time step, the Scheduler receives updates on the energy capacity of the EHWs, the demand, and the wind production, and builds a new scenario tree for *T* periods using this updated information. It then computes a new OPP over the new scenario tree, and again sends the optimal solution to the MFC. We operate in this way because at the start of each day, or whenever an unexpected event causes reality to deviate significantly from the forecast, it is essential to generate a new OPP to prevent too much error accumulation at the level of the MFC. The stochastic optimization model is thus solved repeatedly to calculate the temperature profile that best matches the new reality, and updated instructions are computed and sent by the MFC to the EHWs.

The rest of this paper is organized as follows. In Sect. 2 we introduce the aggregated EWH model and show how to maintain OPP feasibility for the MFC. In Sect. 3 we present the Scheduler. In Sect. 4 we discuss the scenario generation. In Sect. 5 we present a case study and the computational results, and Sect. 6 provides concluding remarks.

## Aggregated EWH model

Our optimization formulation is designed to work in conjunction with the mean field model (Kizilkale and Malhame 2014), where each EWH is modeled by assuming that the reservoir is made up of *n* fully mixed equal volume layers. The cold water inlet is in the bottom layer, and hot water is drawn from the top layer. Our formulation uses an aggregated EWH model by considering a group of homogeneous EWHs as one large thermal battery. We thus model the EWHs as a single-layer large reservoir with a controllable mean water temperature given a sufficiently good approximation of the energy that the EWH population is capable of absorbing as well as of the losses (mostly due to hot water draw events). Thermal energy conservation for the aggregated EWH model, also called the system dynamics, is expressed as:

1 $$\begin{aligned} e_{t+1} = e_{t} + { x }(e_{t}) -\ell (e_{t}). \end{aligned}$$

Here $e_t$ is the stored energy (i.e., the system state) at time step *t*; $x(e_t)$ is the decision variable that represents the quantity of energy injected into the reservoir, which depends on the current system state; and $\ell (e_t)$ is the system loss due to hot water extraction and heat transfer by conduction.

The heat transfer by conduction, denoted $\ell _1(e_t)$, is

2 $$\begin{aligned} \ell _1(e_t) = K A\left( \frac{e_{t}}{{C^p} \rho V} + {N^{\text {ewh}}}({T^L} - {T^{\text {env}}})\right) t \end{aligned}$$

where we have the following quantities are that exogenous inputs: *K* is the thermal conductivity per unit length of EWHs, *A* is the total surface area of all the EWHs, $C^p$ is the hot-water specific heat, $\rho$ is the water density, *V* is the total volume of hot water in the EWHs, ${N^{\text {ewh}}}$ is the number of EWHs in the model, $T^L$ is the inlet water temperature, and ${T^{\text {env}}}$ is the environment temperature.

We model the hot water extraction process as a time process on a finite state space that satisfies the Markov property. Specifically, we adopted the model in Kizilkale and Malhame (2014) where extraction is modeled as a continuous-time Markov chain. It is denoted $\theta _t$, $t \ge 0$, and takes values in $\varTheta = \{1,2,$...$,{\mathfrak {I}}\}$, with the identical infinitesimal generator $\varLambda = \{\lambda _{ij}, i, j,= 1,\ldots ,{\mathfrak {I}}\}$, where each state consists of different drawn water volumes depending on the type of event such as shower or hand washing. In a continuous time Markov chain, the useful information that we use to calculate the probability of a given state is the distribution of the waiting time at every state. The infinitesimal generator parameters, also called transition rate parameters or matrix, describe the rate of movement between states. Hence, $\lambda _{ij}$ describes the rate of transition to state *j* from state *i*. This transition rate is then used to calculate the probability of occurrence of each state, denoted ${\mathfrak {p}}_{i}$ and defined as

3 $$\begin{aligned} \begin{aligned} {\mathfrak {p}}_{i}&= \frac{\varPi _i}{\sum _{k=0}^K \varPi _k} \\ \varPi _0&= 1, \quad \varPi _i = \frac{\lambda _{0,1} \lambda _{1,2} \ldots \lambda _{i-1,i}}{\lambda _{1,0} \lambda _{2,1} \ldots \lambda _{i,i-1}} \text { for } i \ge 1. \end{aligned} \end{aligned}$$

Given this, we can aggregate the losses due to extraction by considering the expected flow of drawn hot water for each type of event *i* as follows:

4 $$\begin{aligned} \ell _2 = \rho {C^p} ({T^{\text {mix}}} - {T^L}) \sum _{i=1}^{{\mathfrak {I}}} {N^{\text {ewh}}} {\mathfrak {p}}_i {\dot{V}}^{\text {mix}}_i. \end{aligned}$$

Here we assume that the end-user mixes hot and cold water together to obtain the desired temperature ${T^{\text {mix}}}$ and the desired flow ${\dot{V}}^{\text {mix}}_k$, with the flow depending on the type of extraction *i*.

**Fig. 1:**
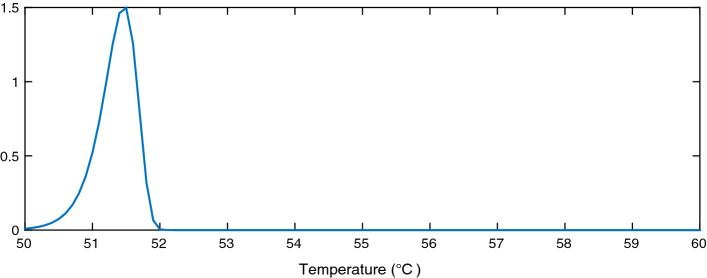
Population distribution near the lower bound

The water temperature must be bounded below to prevent bacterial contamination (especially from Legionella pneumophila, for which the growth potential is almost zero above ${46}^{\circ }\hbox {C}$ (Lacroix 1999)) and bounded above for end-user safety. The zone between these two bounds is the comfort zone that we represent for the aggregate of the EHWs using the constraints:

5 $$\begin{aligned} \begin{aligned} {e^{\text {max}}}&= {N^{\text {ewh}}} \rho V {C^p} ({T^{\text {max}}} - {T^L})&\qquad \forall t,s \\ {e^{\text {min}}}&= {N^{\text {ewh}}} \rho V {C^p} ({T^{\text {min}}} - {T^L})&\qquad \forall t,s \end{aligned} \end{aligned}$$

Note that these expressions are also bounds on the energy that can be stored in the set of EWHs. In other words, the maximum quantity of energy the system is able to absorb, $$ x (e_{t})$$, depends on its mean current state, $$e_t$$, because as this state approaches the upper bound, it is able to accept less energy. Moreover, the EWHs will consume a minimum quantity of energy to prevent the system in aggregate from going below the lower limit of the comfort zone.

To bound the aggregated power consumption of the EWHs, we considered the MFC module of Kizilkale and Malhame (2014) as a black box and used it to calculate the minimum and maximum electric power that the EWH population can consume for all reachable values of $e_t$. Note that not all members of the EWH population reach the same energy level. They are distributed with a certain variance and skewness, where this distribution is not normal because of the comfort zone constraint that trims the tails of the probability density function. Furthermore, the variance and skewness depend on the control. The variance tells us how far the extreme energy states are from the average. We figure out that when the population average is next to the boundaries, the population is more squeezed, in contrary to the case when the population’s mean is far from the boundaries. The former gives us less energy state diversity. The skewness is also important because it shows that the distribution of the EWHs is not symmetric around the mean (i.e., the probability of having EWHs with energy state higher than the average is greater than the probability of having EWHs with less energy). Therefore, we expect that asking the EWH population to reduce its mean temperature is an easier job that increasing it, because the pool size of EWHs which temperature is higher than the mean is larger, hence more flexibility to reduce the mean with minimum individual disturbance in this case. We expect also that with higher diversity, less violation of individual comfort zones will occur.

**Fig. 2:**
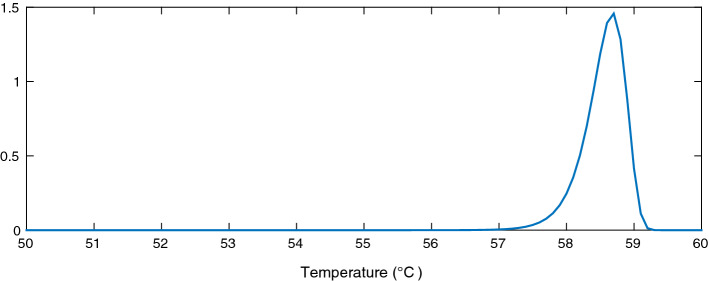
Population distribution near the upper bound

**Fig. 3:**
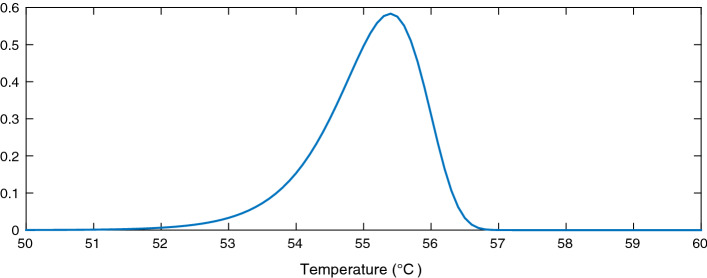
Population distribution in the middle of the comfort zone

We conducted a study of the distribution of the EWH population state around a finite set of system states $e_t$ in order to randomly generate initial EWH states following this precalculated probability density function. For every discrete value of a target energy level $e_t$, we begin the simulation assuming that the energy state of the EWH population follows a normal distribution with mean $\mu$ and variance 1. The tails of the distribution are truncated because we have lower and upper bounds on the inner temperature of the hot water inside the EWH tank, but as shown in Figs. 1, 2 and 3, the area under the probability density function near the bounds is sufficiently small to argue that ignoring the truncation is a fair assumption. The MFC is asked to control the EWH population so that its mean energy state reaches $e_t$. When the population mean converges to $e_t$, we calculate its variance and skewness around $e_t$; we denote this density function by ${f(e_t)}$. Then, for every $e_t$, the state of the EWH population is initialized so that its state distribution follows ${f(e_t)}$. The MFC is next asked to ensure that the mean population temperature is between its lower and upper limits, ${T^{\text {min}}}$ and ${T^{\text {max}}}$. The lower and upper aggregated power consumption bounds can then be calculated. We use this simulation to initialize the energy state of the EHW population, to reflect as much as possible a reasonable initial state of the population, before applying our target control trajectory.

Figures 1, 2 and 3 show the distribution of the thermal energy of the population as its mean moves towards the lower bound, the upper bound, and the middle of the comfort zone. We can see that the thermal energy distribution has a clear negative skew due to the hot water draw events. These result in thermal losses and cause a large portion of the EWHs to have a temperature below the mean population temperature. Furthermore, the distribution variance shrinks as the mean approaches the bounds, which affects the population’s diversity.

**Fig. 4:**
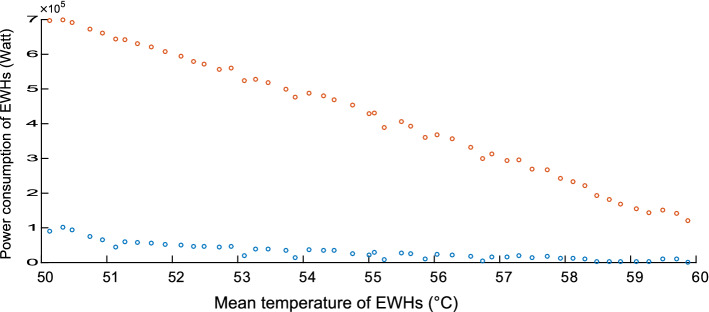
Simulated bounds

**Fig. 5:**
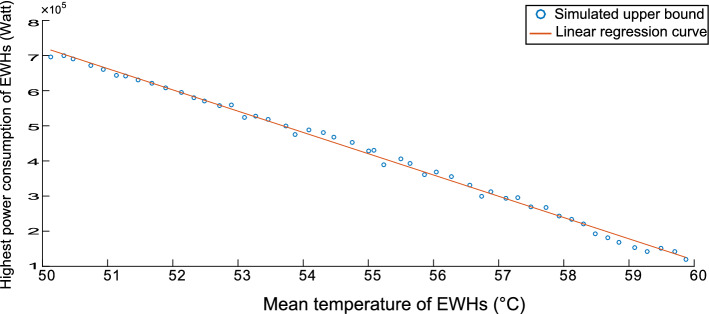
Linear regression for the upper bound

**Fig. 6:**
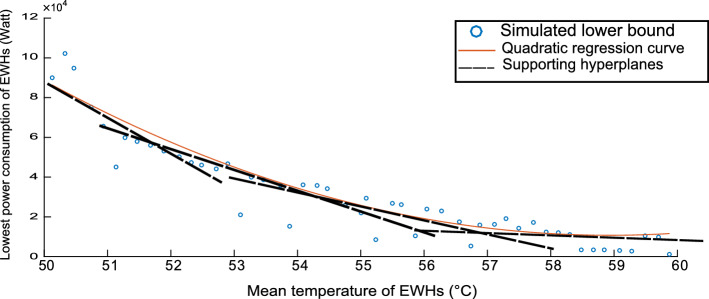
Quadratic regression for the lower bound

Figures 4, 5 and 6 are concerned with bounding the power consumption of the EWHs. Figure 4 shows the simulated results of the minimum and maximum power consumption for 200 EWHs. The feasible region of $$ x (e_{t})$$ is the region between the two monotonically nonincreasing functions. We will need to integrate these bounds in our model. To integrate the upper bound, we apply a linear regression, as shown in Fig. 5. For the lower bound, we use a convex quadratic regression, as shown in Fig. 6; the resulting quadratic function is then outer-approximated by a piecewise linear function formed using supporting hyperplanes.

## Stochastic model

Several optimization techniques have been developed to deal with uncertain parameters, namely stochastic dynamic programming (Bertsekas 2007), robust optimization (Ben-Tal and Nemirovski 2000; Bertsimas et al. 2011; Bertsimas and Sim 2004; Ferreira et al. 2012), chance-constrained optimization (Dorini et al. 2013), and stochastic optimization with recourse (Birge and Louveaux 2011). See (Sahinidis 2004) for a review of optimization under uncertainty in general, and (Pisciella et al. 2015) for a detailed description of different optimization techniques with different levels of granularity for the description of the problem of capacity expansion in power systems.

Uncertain parameters are usually modeled by either distributions or stochastic processes. The former are used when the decision is to be made over a single stage, and the latter when a series of decisions must be taken over multiple stages (Kaut and Wallace 2003). In our case, decisions are taken for hourly time steps in the planning horizon, and the two stochastic processes are the uncontrollable demand and the wind power production.

Stochastic optimization with recourse is our chosen approach. In certain special cases the model is solvable directly with continuous distributions, but most methods require discrete distributions of finite cardinality. The continuous random process must therefore be approximated by a discrete finite set of outcomes in the form of a *scenario tree* that represents the diffusion of stochastic information into the future. The tree represents the multiple stages of the observation of the possible outcomes of the random variables in time. Decisions are taken at different stages depending on the available data at the given stage, and regardless of future observations that are considered uncertain. As more observations are revealed, recourse decisions are made taking the revealed information into account while being consistent with the decisions made in previous stages.

**Fig. 7:**
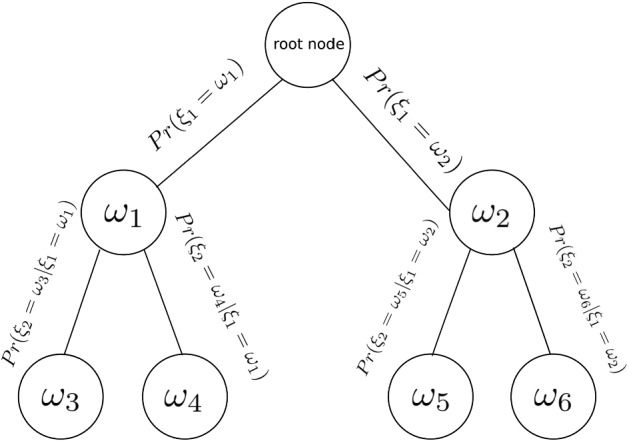
Three-stage scenario tree

Figure 7 illustrates a scenario tree with three stages, where the root node is the value of the discrete stochastic process {$\xi _t$} at $t=0$ and is considered deterministic (i.e., has a probability of occurrence equal to 1). Two possible outcomes at the next stage $t = 1$ are represented by two nodes with values $\omega _1$ and $\omega _2$. Each of these can lead to two possible realizations of the random process, ($\omega _3,\omega _4$) and ($\omega _5,\omega _6$) respectively, with their conditional probabilities shown on the arcs. The number of stages in a scenario tree does not necessarily reflect the number of time steps in the optimization problem but rather the number of times that new observations of the random process are made. The larger the scenario tree, the better the representation of the uncertain stochastic process, but also the larger the optimization problem.

### Mathematical model

The objective of the Scheduler is to even out the net demand curve. This curve is denoted $p_n$ and is equal to the demand remaining (in MW) after the renewable production has been dispatched. We compute the optimal electric power consumption (in MWh) of the controllable EWH load of EWHs, denoted $x_n(e_n)$, so that valleys are filled and peaks are lowered while respecting the end-user comfort. The net demand is

6 $$\begin{aligned} p_n = d(\omega _n) - r(\omega _n) + \frac{ x_{{\widehat{n}}}(e_{{\widehat{n}}}) }{\varDelta t} \end{aligned}$$

where $d(\omega _n)$ and $r(\omega _n)$ are respectively the observed values of the uncontrollable demand (in MW) and the wind power production (in MW) at node $n \in {1,\ldots ,N}$ of the scenario tree with *N* nodes, and $\varDelta t$ is the discrete time step. To respect the nonanticipativity conditions, the decision $x_{{\widehat{n}}}(e_{{\widehat{n}}})$ is taken at the parent node of node *n* in the scenario tree, denoted ${\widehat{n}}$, before we observe the realization of $d(\omega _n)$ and $r(\omega _n)$. At the root node *d* and *r* are deterministic (observed) parameters, where *d* is the actual total demand, and *r* is the actual wind power.

Evening out the net demand curve involves minimizing the absolute value of the difference in the net demands at two consecutive nodes in the scenario tree multiplied by their probability of occurrence:

7 $$\begin{aligned} \min \quad \sum _{n=0}^{N} {{\mathbb {P}}_n} z_n \end{aligned}$$

where ${{\mathbb {P}}_n}$ is the marginal probability of the occurrence of node *n*, independent from its parent node ${\widehat{n}}$, and $z_n$ is the absolute value of the difference between the net demands at node *n* and its parent node ${\widehat{n}}$. We can model this absolute value using the following linear formulation:

8 $$\begin{aligned} z_n \ge p_n - p_{{\widehat{n}}}, \quad z_n \ge p_{{\widehat{n}}} - p_n. \end{aligned}$$

The resulting stochastic optimization model is:

$$\begin{aligned} \min _{e, x, z \in {\mathbb {R}}^n} \quad \quad&\sum _{n=0}^{N} {{\mathbb {P}}_n} z_n \\ \text {s.t.} \quad z_n&\ge p_n - p_{{\widehat{n}}} \quad \forall n \\ z_n&\ge p_{{\widehat{n}}} - p_n \quad \forall n \\ p_n&= d(\omega _n) - r(\omega _n) + \frac{ x_{{\widehat{n}}}(e_{{\widehat{n}}}) }{\varDelta t} \quad \forall n \\ e_{n}&= e_{{\widehat{n}}} + {x_{{\widehat{n}}}}(e_{{\widehat{n}}}) -\ell (e_{{\widehat{n}}}) \quad \forall n \\ \ell _1(e_n)&= K A\left( \frac{e_{n}}{{C^p} \rho V} + {N^{\text {ewh}}}({T^L} - {T^{\text {env}}})\right) \varDelta t \quad \forall n \\ \ell _2&= \rho {C^p} ({T^{\text {mix}}} - {T^L}) \sum _{i=1}^{{\mathfrak {I}}} {N^{\text {ewh}}} {\mathfrak {p}}_i {\dot{V}}^{\text {mix}}_i \\ \ell (e_{{\widehat{n}}})&= \ell _1(e_{{\widehat{n}}}) + \ell _2 \quad \forall n \\ e_{o}&= N \rho V {C^p} (T_{init}-{T^L}) \\ e_{n}&\le {N^{\text {ewh}}} \rho V {C^p} (T_{\max } - {T^L}) \quad \forall n \\ e_{n}&\ge {N^{\text {ewh}}} \rho V {C^p} (T_{\min } - {T^L}) \quad \forall n \\ x_n(e_{n})&\le A_1 (e_{n}) + B_1 \quad \forall n \\ x_n(e_{n})&\ge \underline{ Q }(e^i) + \underline{ \mathring{Q} }(e^i) ( e_n - e^i ) \quad \forall n,i \\ x_n(e_{n})&\ge 0 \quad \forall n \\ e_{n}&\ge 0 \quad \forall n \end{aligned}$$

Here we have included the bounds on $x_n(e_{n})$ from the regression approximation, and also the last two inequalities that require the non-negativity of the energy injected and of the stored energy.

## Scenario generation

In this section we describe the generation of the multistage scenario tree for our stochastic optimization problem. Artelys, a company specializing in optimization, decision support, and modeling, cooperated with us by developing a load forecast model trained over a set of historical data for power consumption from 2012 to 2014. The data were provided by the supervisory control and data acquisition (SCADA) section of the Coopérative Régionale d’Electricité de Saint-Jean-Baptiste de Rouville (CoopSJB). The CoopSJB data are collected from five distribution substations in Mont Saint-Hilaire, a suburb of Montreal, for 6819 houses. They are normalized to obtain the mean power consumption per house. The forecast model takes as inputs the hourly wind speed and temperature of day $d_k$ and the hourly forecast for day $d_{k+1}$, and it outputs the hourly load demand forecast for $d_{k+1}$. Multiple load demand curves are generated using wind speed and temperature ensemble forecasts provided by Environment Canada, which releases each day a set of 22 forecasts for the next 144 h. The uncontrollable demand component is obtained by computing an estimate of the hourly EWH consumption using the data from CoopSJB, and subtracting this estimate from the total demand. Wind power scenarios are generated from the wind speed ensemble forecasts using the approach proposed in Tammam et al. (2015). Every uncontrollable load demand scenario is coupled with its corresponding wind power scenario resulting from the same wind speed forecast.

We construct a collection of 22 scenarios with 24 nodes per scenario for the 24-h horizon in the following way. Each node has two (hourly) values: uncontrollable demand and wind power production. We first construct a so-called “comb” scenario tree with what amounts to two stages: the first stage corresponds to the root, and the second stage is composed of 22 possible branches from the root. Each of those branches covers all the remaining time periods in a deterministic fashion. It is common to use this kind of two-stage structure to describe complex systems evolving over several periods: Except for the first period, all periods are lumped into the second stage. In the context here, we chose to use this deterministic-equivalent approach because it is compatible with the application of this model jointly with the MFC. Note that while it is well known that this approach does not generally scale well for large scenario trees, we do not experience this scaling problem because we restrict ourselves to “comb” scenario trees.

However, as a consequence, this tree structure does not capture future uncertainty well because it does not possess the non-anticipativity property. To overcome this shortcoming, we apply the forward tree construction algorithm of Heitsch and Römisch (2007) to obtain a tree with only 22 leaves but that is non-anticipative. While this scenario tree represents only a small fraction of the possible outcomes over a 24-h period, it still forces decisions to account for uncertainty.

## Case study and results

We performed a case study in the context of the smartDESC (smart Distributed Energy Storage Controller) project. The goal was to obtain a proof of concept for a hierarchical control architecture managing the power consumption of dispersed energy storage devices in the electric grid. The controller aimed to mitigate the variability caused mainly by the increasing penetration of renewable energy resources.

We note that we have not provided direct comparisons with other works in the literature. There are two reasons for this. First, most of the other approaches mentioned in Sect. 1 are deterministic. Second, many of the approaches in the literature are centralized methods that address the whole process, unlike ours that is (a) designed specifically to work together with a request dispatch method that is completely separate, and for our purposes is a “black box” (the MFC), and (b) centralized methods are highly impractical because of the high communication requirements, and indeed the architecture of SmartDESC (Sirois et al. 2017) was motivated by the desire to avoid this.

### Results

The aggregated model used in this case study represents a population of 200 EWHs; but the curves in this section are averaged over 200 houses. The planning horizon is 24 h with hourly time steps. We use a rolling horizon: at every time step *t*, a new scenario tree, denoted $\{\xi _t\}_{t \in T}$, is created based on the available wind speed and demand forecasts. We retain only the optimal solution $x_{0,t}^*$ at the root node $\xi _{0,t}$ of the tree, where the root node contains the values of total demand and wind production observed at the previous time step $t-1$.

We scale the wind power production scenarios so that the average of their maximum values over the planning horizon is equal to 10% of the average value of the maximum uncontrollable demand over the horizon. We did this to test the load curve reduction the system can achieve when the maximum wind power production is on average equal to 10% of the uncontrollable demand.

**Fig. 8:**
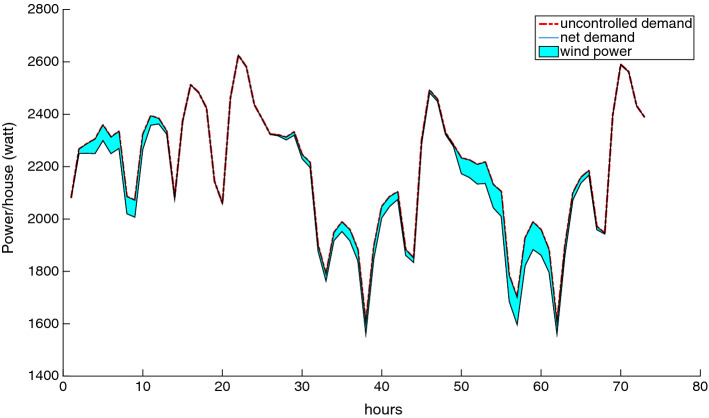
Case study with minimum wind power

**Fig. 9:**
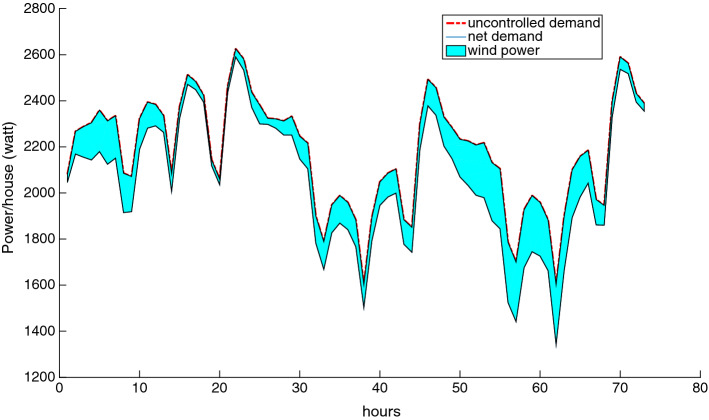
Case study with average wind power

**Fig. 10:**
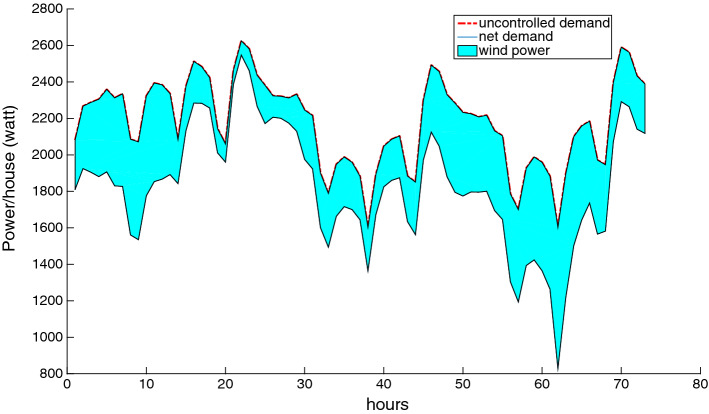
Case study with maximum wind power

As previously mentioned, the root node of the scenario tree $\{\xi _t\}_{t \in T}$ contains the actual realization of the total demand and wind power. Therefore, we must construct a deterministic observation of these two parameters over the planning horizon. We present three cases in which the mean uncontrollable demand forecast is considered as the observed uncontrollable demand, and this demand is coupled with three different wind power observations: minimum, average, and maximum power. This is depicted in Figs. 8, 9 and 10, where the upper curve is the uncontrolled demand, the lower curve is the net demand, and the region in between represents the magnitude of the wind power. We see that, in spite of the wind fluctuations, our approach can absorb this intermittence to ensure the balance between supply and demand.

**Fig. 11:**
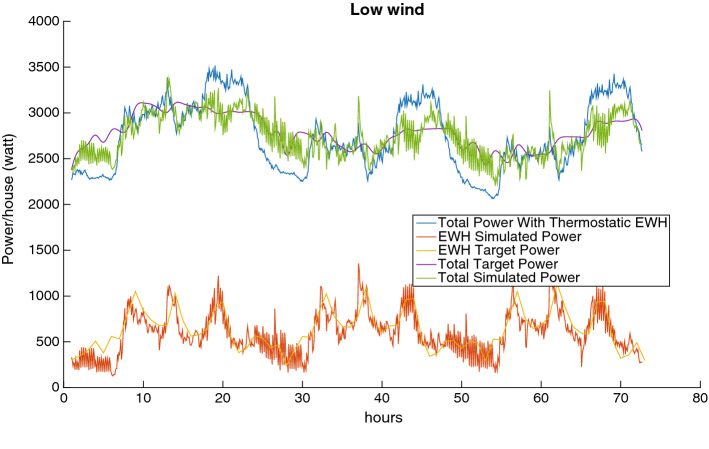
Load reduction with low wind power

**Fig. 12:**
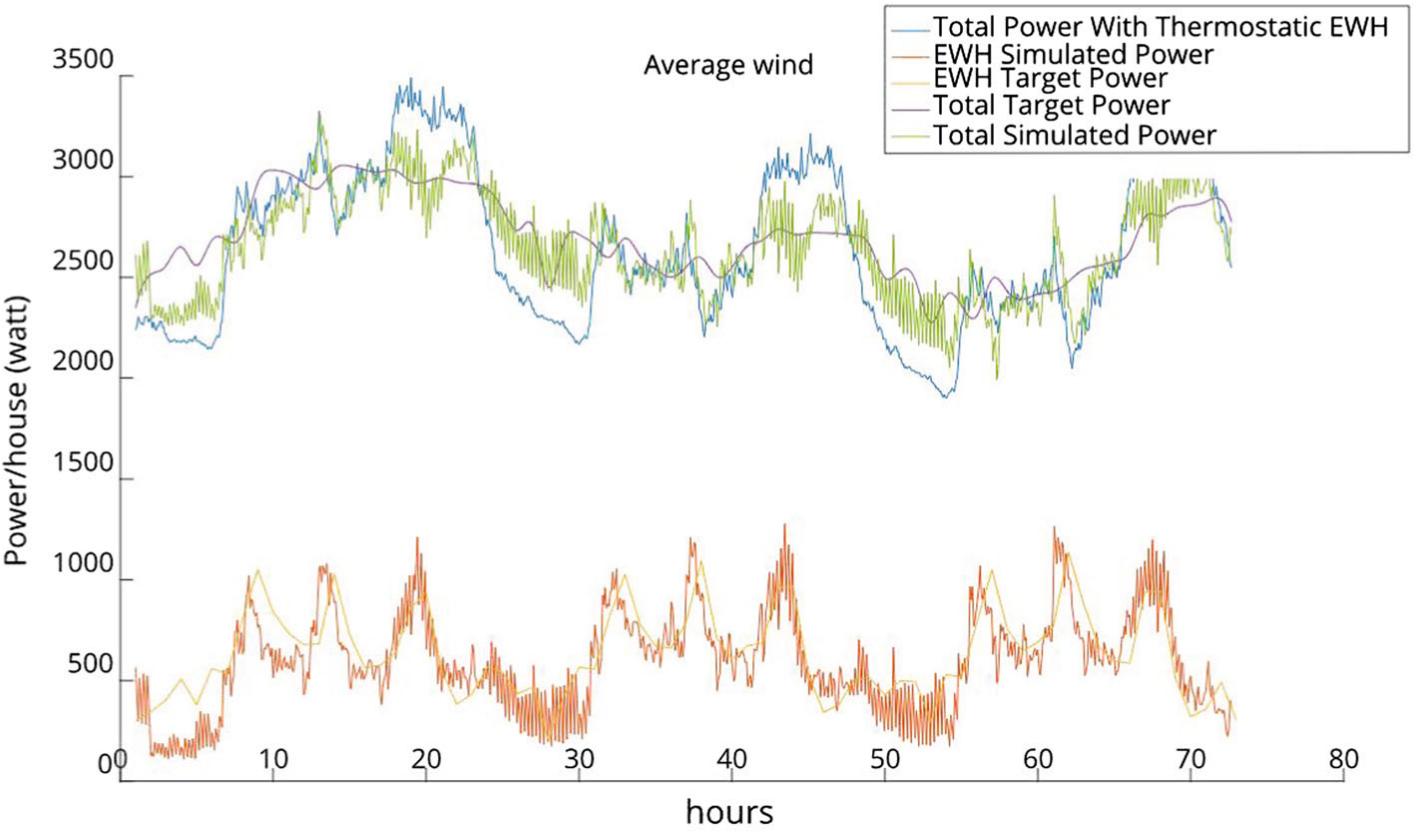
Load reduction with average wind power

**Fig. 13:**
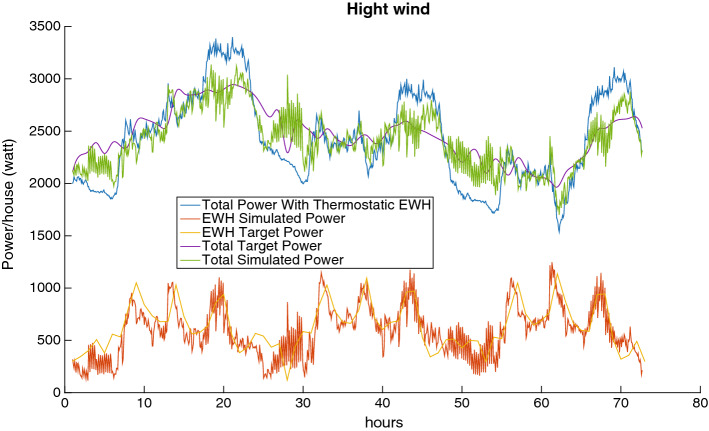
Load reduction with high wind power

Figures 11, 12 and 13 depict the load reduction curves with low, average, and high wind penetration. There are six curves for each of these cases of wind penetration:

- The EHW Target Power is the target given by the Scheduler to the MFC.
- The EWH Simulated Power is the level of consumption that the MFC is able to achieve (given the EHW target power).
- The Total Target Power is the resulting total power that the Scheduler expects to achieve by proposing the EWH target power.
- The Total Simulated Power is the total power that the MFC is able to achieve.
- Finally, the Total Power With Thermostatic EWH is the power level before we intervene, i.e., it is the power curve before our balancing is applied.

From these figures, we can make the following observations:

- The target power consumption profile generated by the Scheduler is generally feasible with respect to the MFC, i.e., the EWH simulated power curve and the EWH target power curve almost overlap.
- The total simulated power generally fluctuates less than the total power before we intervene, i.e., the green curve achieves significant curtailment of the peaks of the blue curve, and moreover the green curve also fills several of the low valleys of the blue curve.

The outcomes observed in Figs. 11 to 13 can also be quantified by the results in Table 1. This table shows the peak reduction resulting from the direct control of the power consumption of the EWH population as a percentage of the load peak in the thermostatic control mode (third column); the reduction in demand variance as a percentage of the demand with the thermostatic control of EWHs (fourth column); and the computational time required to solve the rolling-horizon problem (last column). The reduction in demand variance is an estimate of how well the net demand curve is evened out.

Clearly, as wind power is added to the grid, the variation in the net demand increases, and it is more challenging to manage the EWH load to reduce the demand fluctuation. This can be seen by comparing the net demand variation reductions for 10% and 20% wind penetration.

**Table 1:** Comparison of three cases of wind blow for 10% and 20% of wind power penetration over three days

Wind penetration (%)	Wind	Peak reduction (%)	Net demand variation reduction (%)	Time (s)
10	High	6.68	46.40	21.535
	Average	7.85	49.42	21.471
	Low	7.84	50.82	21.454
20	High	6.51	32.82	22.674
	Average	8.57	42.60	21.849
	Low	8.46	45.16	22.544

**Fig. 14:**
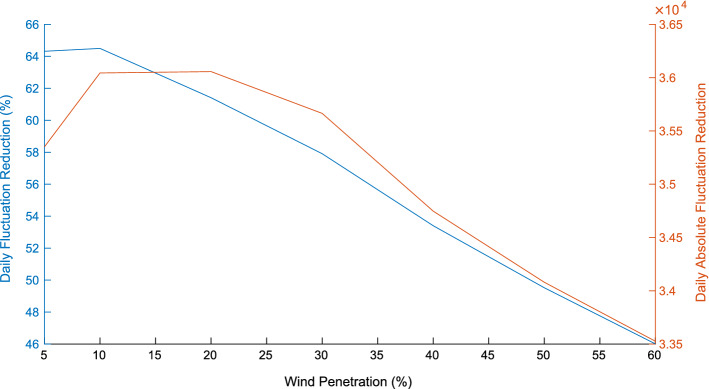
Reduction of daily fluctuation versus wind penetration

**Fig. 15:**
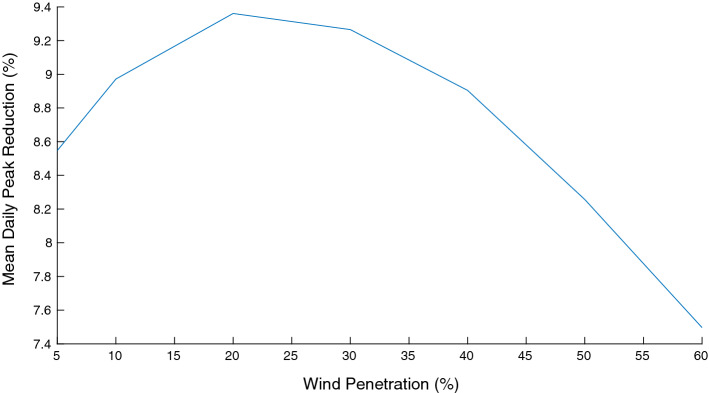
Mean daily peak reduction versus wind penetration

Two other observations are important. First, Fig. 14 shows that when the wind power penetration to the grid increases, the reduction of the net demand load curve fluctuation is smaller. This is because of the limited ability of EWHs to absorb the wind power variability. On the other hand, Fig. 15 shows how this increase in wind power permits the operator to reduce the peak of the load curve, although eventually this gain in peak reduction decreases as the penetration of renewables increases. These results were obtained with a participation of 100% of the EWH population in the demand response program.

## Conclusion

We have proposed a multistage stochastic optimization model for load reduction in the presence of renewable resources attached to the grid, by means of the storage capacity of residential EWHs. This model is a part of the smartDESC project. The project provides a hierarchical control architecture that manages dispersed devices locally and more efficiently while achieving the global goals of peak reduction and net demand flattening. The model shows the impact of renewable resources on the variability of the net demand curve. This gives the system operator information about how much renewable power it can afford while maintaining a stable and serviceable demand.

## Indices

*t*: Time steps
*n*: Nodes of the scenario tree
*i*, *j*: States of the Markov chain / types of water extraction

## Parameters

*K*: Thermal conductivity per unit length of EWHs
*A*: Total surface area of all the EWHs
$C^p$: Hot-water specific heat
$$\rho $$: Water density
*V*: Total volume of hot water in the EWHs
${N^{\text {ewh}}}$: Number of EWHs
$T^L$: Inlet water temperature
$$T^{\text {env}}$$: Environment temperature
$\varTheta = \{1,2,$...$,{\mathfrak {I}}\}$: Set of water extraction values for the Markov chain $\theta _t$
$\varLambda = \{\lambda _{ij}, i, j,= 1,\ldots ,{\mathfrak {I}}\}$: Infinitesimal generator for the Markov chain $\theta _t$
${\mathfrak {p}}_{i}$: Probability of occurrence of each state of the Markov chain
${T^{\text {mix}}}$: Desired temperature for the end-user of hot water
${\dot{V}}^{\text {mix}}_i$: Desired flow of water for the end-user for extraction of type *i*
${e^{\text {min}}}$: Minimum amount of energy that can be stored in the EWHs
${e^{\text {max}}}$: Maximum amount of energy that can be stored in the EWHs
${T^{\text {min}}}$: Lower limit of the temperature of the population of EWHs
${T^{\text {max}}}$: Upper limit of the temperature of the population of EWHs
*N*: Number of nodes in the scenario tree
*T*: Number of time periods
$\varDelta t$: Discrete time step
$$\{\xi _t\}_{t \in T}$$: Scenario tree
$d(\omega _n)$: Value of the uncontrollable demand at node *n* of the scenario tree
$r(\omega _n)$: Wind power production at node *n* of the scenario tree
${\widehat{n}}$: Parent node of node *n* in the scenario tree
${{\mathbb {P}}_n}$: Marginal probability of the occurrence of node *n*

## Decision variables

$e_t$: Energy stored in the system at time step *t*
$x(e_t)$: Quantity of energy injected into the EHWs at time step *t*
$p_n$: Net demand curve for the mean field controller

## Other variables

$\ell _1(e_t)$: Heat transfer by conduction
$\ell (e_t)$: Energy loss due to hot water extraction and heat transfer by conduction
$\theta _t$, $t \ge 0$: Continuous-time Markov chain of water extractions
${f(e_t)}$: Density function of $e_t$
